# An international data set for CMML validates prognostic scoring systems and demonstrates a need for novel prognostication strategies

**DOI:** 10.1038/bcj.2015.53

**Published:** 2015-07-31

**Authors:** E Padron, G Garcia-Manero, M M Patnaik, R Itzykson, T Lasho, A Nazha, R K Rampal, M E Sanchez, E Jabbour, N H Al Ali, Z Thompson, S Colla, P Fenaux, H M Kantarjian, S Killick, M A Sekeres, A F List, F Onida, R S Komrokji, A Tefferi, E Solary

**Affiliations:** 1 Department of Malignant Hematology, H Lee Moffitt Cancer Center and Research Institute, Tampa, FL, USA; 2 Department of Leukemia, The University of Texas MD Anderson Cancer Center, Houston, TX, USA; 3 Division of Hematology, Mayo Clinic, Rochester, MN, USA; 4 Service d'Hematologie, Hopital Avicenne, Bobigny, France; 5 Leukemia Program, Department of Hematology and Medical Oncology, Cleveland Clinic Taussig Cancer Institute, Cleveland Clinic, Cleveland, OH, USA; 6 Leukemia Service, Department of Medicine, Memorial Sloan Kettering Cancer Center, New York, NY, USA; 7 Department of Biostatistics, H Lee Moffitt Cancer Center and Research Institute, Tampa, FL, USA; 8 Service d'Hématologie Séniors, Hôpital St Louis, Université Paris 7, Paris, France; 9 Department of Haematology, Royal Bournemouth Hospital, Bournemouth, UK; 10 Fondazione IRCCS Ca' Granda Ospedale Maggiore Policlinico, University of Milan, Milan, Italy; 11 Gustave Roussy, INSERM U1170, Villejuif, France

## Abstract

Since its reclassification as a distinct disease entity, clinical research efforts have attempted to establish baseline characteristics and prognostic scoring systems for chronic myelomonocytic leukemia (CMML). Although existing data for baseline characteristics and CMML prognostication have been robustly developed and externally validated, these results have been limited by the small size of single-institution cohorts. We developed an international CMML data set that included 1832 cases across eight centers to establish the frequency of key clinical characteristics. Of note, we found that the majority of CMML patients were classified as World Health Organization CMML-1 and that a 7.5% bone marrow blast cut-point may discriminate prognosis with higher resolution in comparison with the existing 10%. We additionally interrogated existing CMML prognostic models and found that they are all valid and have comparable performance but are vulnerable to upstaging. Using random forest survival analysis for variable discovery, we demonstrated that the prognostic power of clinical variables alone is limited. Last, we confirmed the independent prognostic relevance of *ASXL1* gene mutations and identified the novel adverse prognostic impact imparted by *CBL* mutations. Our data suggest that combinations of clinical and molecular information may be required to improve the accuracy of current CMML prognostication.

## Introduction

Chronic myelomonocytic leukemia (CMML) is a heterogeneous malignancy characterized by peripheral blood monocytosis and a propensity for acute myeloid leukemia transformation.^1, 2^ Its clinical heterogeneity is broadly captured by the French–American–British group, which defines myelodysplastic syndrome (MDS)-CMML and myeloproliferative neoplasm (MPN)-CMML based on the latter having a white blood cell count >13 × 10^3^ cells per dl.^3^ The World Health Organization (WHO) reclassified CMML as a distinct disease entity under the MDS/MPN designation in 2008.^4^ This reclassification has been substantiated by recent advances in the genetic and molecular pathogenesis of CMML, which has confirmed CMML to be biologically distinct from MDS.^5, 6, 7, 8^ Since its reclassification, clinical research efforts have begun to delineate CMML-specific tools and therapeutics. Several prognostic models derived from smaller data sets have been developed to stratify CMML patients into groups that are predictive for overall survival (OS).^9, 10, 11, 12, 13, 14, 15, 16, 17, 18^ However, the validity of these models in a large international data set has never been investigated, and a consensus is not yet present that would standardize risk stratification.

The incidence of CMML is estimated at 0.4 per 100 000 based on several large epidemiologic studies.^19, 20^ Given the apparent low incidence of CMML and its broad range complexity, detailed baseline characteristics describing the clinical heterogeneity have not been reported in a large data set. To examine CMML baseline characteristics and test the prognostic significance of clinical and genetic variables, as well as the relative power of existing prognostic models with sufficient resolution, we constructed a large international CMML database that merged CMML registries from eight tertiary cancer centers across three different countries.

The aims of this study were to establish a large disease-specific data set capable of discerning independent covariates predictive of disease behavior. Using this data set, we explored and annotated the frequencies of clinically relevant disease characteristics. We additionally attempted to validate prognostic models used in CMML clinical practice and determined their relative statistical power within our data set, as well as explored the possibility of constructing a novel model that would increase prognostic capacity in CMML. Last, we examined the prognostic significance of the most frequent mutations in CMML to determine if their incorporation would better refine disease prognostication.

## Materials and methods

Participating centers were identified via the International Working Group for MPNs and the Evans Foundation MDS Clinical Consortium. Data were abstracted from the first visit at each institution and deposited for central data review at the Moffitt Cancer Center. Internal Review Board approval was obtained at each respective institution. Manual central review of cases was performed to ensure data quality before analysis. Data curation and merging were performed to ensure that (1) data elements were uniformly recoded in all spreadsheets for accuracy and consistency; (2) data were centrally transformed into categorical variables for analysis; (3) descriptive cytogenetic information was uniformly categorized according to the International Prognostic Scoring System (IPSS), Revised (R)-IPSS, Mayo and Spanish prognostic models (CPSS);^10, 11, 12^ (4) calculated scores for the different prognostic models in CMML were centrally reviewed and concordant with the methodology in their respective publications; and (5) baseline data were reflective of CMML that was strictly defined according to the WHO criteria. The primary objective of this study was to establish an international CMML data set and validate the above models calculated at the time of presentation to each center.

We validated and performed a detailed statistical comparison between the IPSS,^11^ R-IPSS,^10^ Global MD Anderson Scoring System,^18^ MD Anderson Prognostic Score,^17^ Dusseldorf Score (DS),^13^ Mayo,^9^ and CPSS.^12^ All prognostic models were calculated as previously described. The Kaplan–Meier (KM) method was used to estimate the median OS and leukemia-free survival (LFS) and the log-rank test was used to compare KM survival estimates with SPSS version 21.0 (IBM Corp., Armonk, NY, USA). Random forest survival and receiver operator characteristic (ROC) analyses were carried out with R. Comparison of relative statistical power was performed with the Harrell's concordance index (C-index) and the area under the curve (AUC) of the ROC. Patients who received allogeneic stem cell transplant (*n*=129) were censored from all survival analysis.

Genetic data were retrospectively collected from each institution. Although raw data were not centrally acquired, the genetic data merged in this data set were previously published from their respective cohorts or generated in a CLIA (Clinical Laboratories Improvement Act of 1988) environment using next-generation sequencing technology as part of the patient's permanent medical record. Published genetic data were generated by both Sanger and next-generation sequencing with amplicon-based target enrichment. The methods for sequencing and bioinformatics analysis have been previously published.^7, 14, 15, 16, 21^

### Role of the funding source

The study sponsors had no role in the study design; no role in the collection, analysis and interpretation of data; no role in the writing of the report; and no role in the decision to submit the paper for publication.

## Results

### Baseline characteristics

Between July 1981 and June 2014, 1832 CMML patients were captured in the international CMML database. Each deposited CMML case contained up to 70 discrete data elements that could include genetic information. The median age at diagnosis was 70 (16–93) years, with a male (67%) predominance. Most patients were evenly subcategorized as MPN-CMML (49.8%) versus MDS-CMML (50.2%) by the French–American–British criteria. Splenomegaly was demonstrable in 25% of all cases. Most patients had favorable cytogenetics by IPSS (73%), R-IPSS (71%), CPSS (71%) and Mayo (71%) classification schemas. The mean bone marrow (BM) blast percentage was 5.6 cellsx10^3^/dl (0–19), and mean monocyte count was 4.85x10^3^/dl (1–120). Surprisingly, the majority of patients had CMML-1 (79.9% vs 20.1%) by the WHO classification schema. Given that the vast majority of patients were classified under CMML-1, we wondered whether our data set supported this cut-point as a major discriminator of prognosis. To test this, we grouped our data according to a BM blast percentage of <5, 5–9 and ⩾10%. Although we were able to confirm the prognostic significance of a blast percentage of ⩾10% by KM survival analysis, we were also able to demonstrate that those cases with 5–9% BM blasts had a median OS comparable to those with ⩾10% (Supplementary Figure S1a). We next attempted to identify the most appropriate blast cut-point using a survival regression tree approach.^22^ By testing every possible cut-point between 3% and 15% BM blasts, this method calculated an estimated relative event rate for each group and determined that 7.5% is the optimal cut-point based on a likelihood ratio test splitting criteria.^23^ To confirm 7.5% as an optimal cut-point, the log-rank tests were calculated at every possible cut-point from 3 to 15%. This confirmed that the cut-point of 7.5% had the highest log-rank test statistic (Supplementary Figure S1b).

Our data suggest that a cut-point of 7.5% blasts may be a more appropriate discriminator of prognosis in CMML. The median OS of the entire data set was 31 (22–64) months. At last follow-up, 1116 deaths (61%) were recorded and 380 leukemia transformations (21%) were observed. An extended description of baseline characteristics and their differences among contributing centers are present in Supplementary Table S1.

### Analysis of existing prognostic models

**Figure 1:**
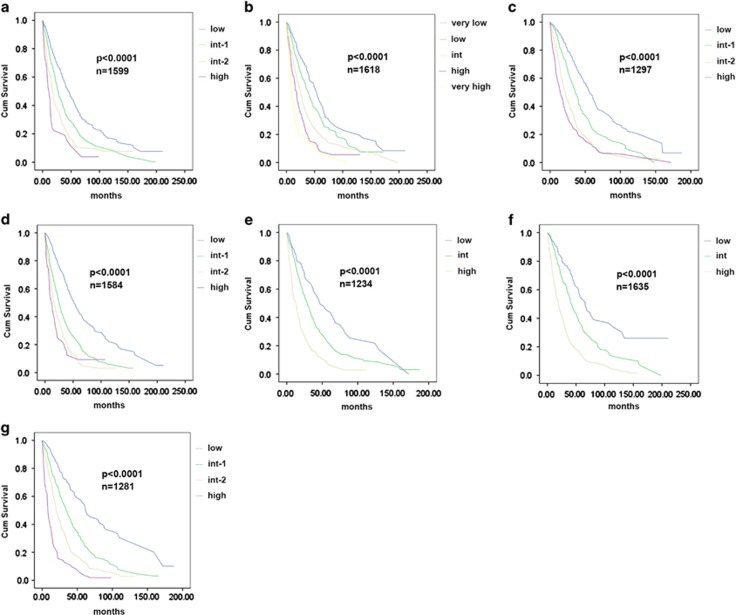
KM survival analysis of seven existing CMML prognostic models. KM survival analysis of (**a**) IPSS, (**b**) R-IPSS, (**c**) MD Anderson Scoring System, (**d**) MD Anderson Prognostic Score, (**e**) DUSS, (**f**) MAYO and (**g**) CPSS. Number of evaluable cases for each model and *P*-value from log-rank test is shown.

**Figure 2:**
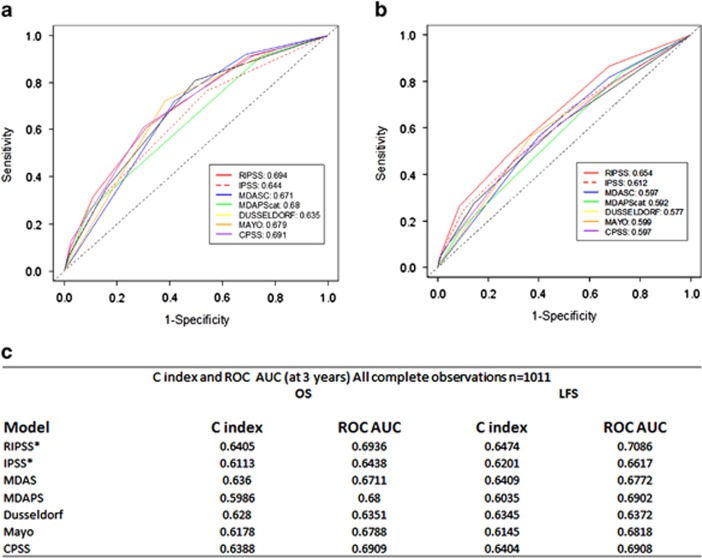
Relative prognostic power of existing CMML models using the entire cohort. ROC curves of all clinical models tested in 1011 evaluable cases in shown for OS (**a**) and LFS (**b**) at 36 months. A comparison between the AUC of the ROC curves and the Harrell's C-index is shown in (**c**). *P<0.05 when comparing AUC of R-IPSS to IPSS.

**Figure 3:**
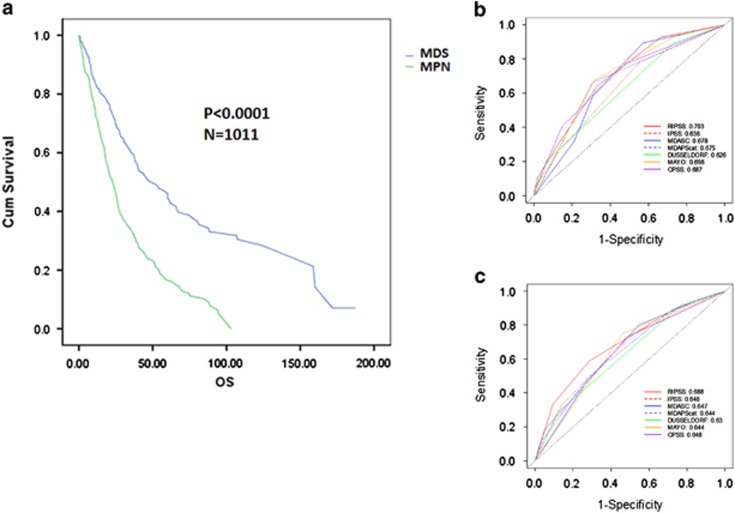
Relative prognostic power of existing CMML models parsed by MDS-CMML and MPN-CMML. The OS of our international database parsed by MDS-CMML and MPN-CMML (**a**). The ROC curves of all clinical models tested for MDS-CMML (**b**) and MPN-CMML (**c**) is shown for OS at 36 months.

To confirm that existing CMML prognostic models were valid in our merged database, we calculated the prognostic score for the IPSS (*n*=1599), R-IPSS (*n*=1618), MD Anderson Scoring System (*n*=1297), MD Anderson Prognostic Score (*n*=1584), Dusseldorf Score (*n*=1234), Mayo (*n*=1653) and CPSS (*n*=1281) for each evaluable case. All tested prognostic models were valid and able to predict OS by the KM method and the log-rank test (*P*<0.0001) (Figure 1). Next, we compared the relative model performance using 1013 complete cases with sufficient data to calculate all risk models using ROC curves and their AUC. ROC curves were calculated for OS at 36 months. The C-index, which evaluates prognostic power across time points, was also used to orthogonally validate the relative prognostic power of each model. The R-IPSS model had the highest AUC (0.694), whereas the Dusseldorf Score model had the lowest (0.635). The difference in AUC between the R-IPSS, IPSS and Dusseldorf Score models was statistically significant (*P*=0.003), whereas there was no significant difference between any other models tested, suggesting that the majority of models were comparable (Figure 2). Because there was a significant survival difference between MDS-CMML and MPN-CMML, suggesting discordant disease behavior, we parsed our cases by French–American–British category to determine whether a specific model would be superior when considering only these subgroups. However, calculating the AUC of the ROC and the C-index again could not identify a statistically superior model (Figure 3).

Last, we reasoned that a fundamental task of cancer prognostic models is to identify *bona fide* low-risk disease cases. It is critical that these cases behave indolently because low-risk cases are often monitored without therapeutic intervention. We therefore determined which CMML models were most vulnerable to reclassification from low risk to higher risk by isolating all respective low-risk cases and applying competing models to identify low-risk CMML cases that were ‘upstaged' to higher risk. We calculated a vulnerability score defined by the number of models able to upstage low-risk disease in >15% of cases. Although the Mayo and MD Anderson Scoring System scores were least vulnerable to upstaging by other models using this metric, all low-risk cases were vulnerable to upstaging (Supplementary Table S2).

### Random forest survival analysis

**Figure 4:**
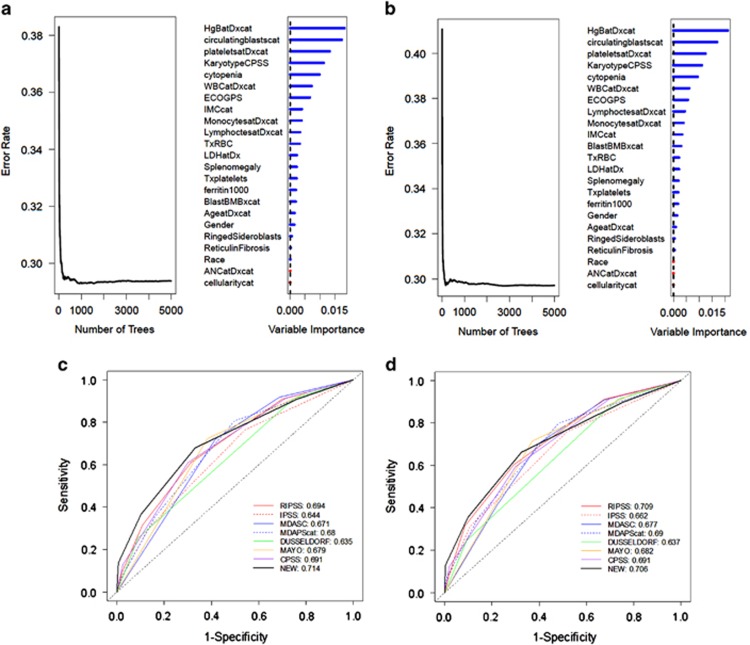
Random forest survival analysis generates a novel CMML model that is comparable to existing models. The results of the random forest survival analysis for OS (**a**) and LFS (**b**) are shown. The ROC curves for all clinical models, including the new model generated using the variables discovered with random forest analysis is shown for OS (**c**) and LFS (**d**).

All existing CMML clinical prognostic models tested were comparable and derived using a Cox proportional hazard regression and multivariate analyses approach. To determine if a novel strategy of prognostic variable discovery could yield an improved model, we performed a random forest survival analysis. This approach iteratively bifurcates the data set based on each variable and, after over 5000 permutations, determines variables of highest importance based on their ability to successfully bifurcate CMML cases based on our desired end point of OS and LFS.^24^ With this approach, 23 categorical variables were considered and ranked by importance, as shown in Figure 4. The top four variables for both OS and LFS were hemoglobin level <11 g/dl, the presence of circulating blasts, a platelet count of <100 × 10^3^/dl and an adverse karyotype as defined by the CPSS. These variables were each assigned one point, and a new prognostic scoring system was devised that stratified our CMML cases into low risk (0 points), intermediate risk (1–2 points) and high risk (3-4 points). KM survival analysis and log-rank test within our database demonstrated a significant OS difference among these risk groups at not reached (95% confidence interval: 53.6–79.2), 35.1 months (95% confidence interval: 32.6–38.4) and 13.8 months (95% confidence interval: 11.7–15.4), respectively (P<0.0001). These results, and the statistically significant differences in LFS among groups (*P*<0.0001), are shown in Supplementary Figure S2. Next, we tested the relative prognostic power of this novel model against other existing CMML models and found that it had the highest AUC at 0.714 for OS and second highest AUC for LFS at 0.709 (similar results for C-index). However, the difference in AUC between our novel model and existing models was not statistically significant, despite being compared within the data set for which the new model was developed (Figure 4).

### Impact of genetic data on prognosis

**Figure 5:**
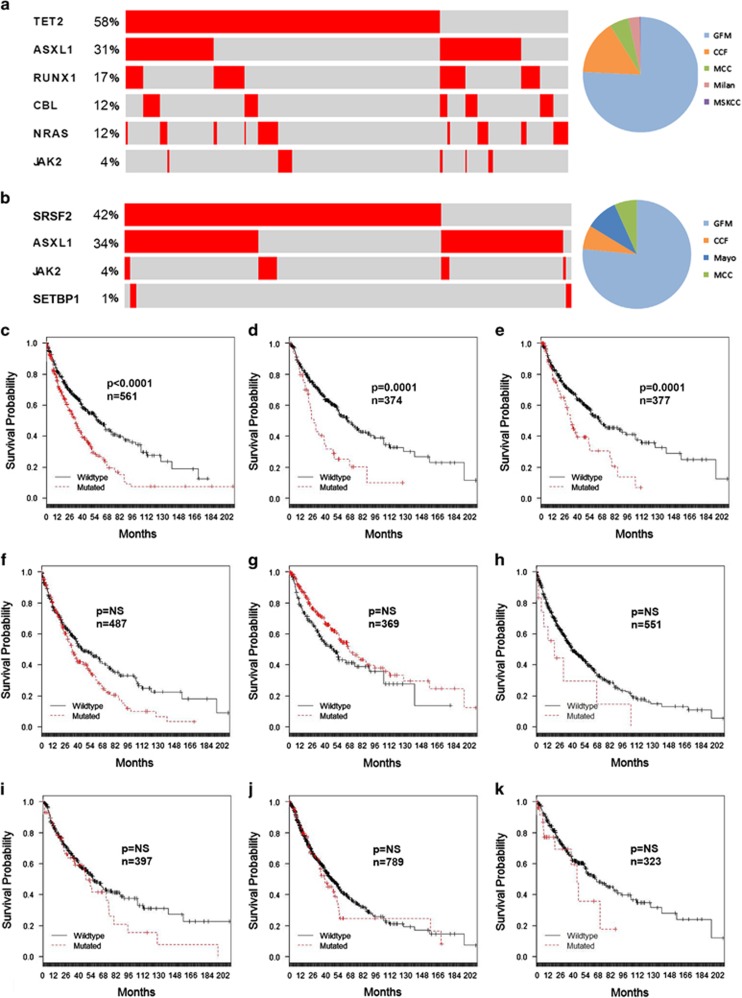
Prognostic significance of genetic data in the international CMML database. The frequency and distribution of mutations is shown using the cbioportal oncoprinter for two clinically relevant subgroups and the number of cases contributed from each center (**a** and **b**). The KM survival analysis for (**c**) *ASXL1*, (**d**) *CBL*, (**e**) *RUNX1*, (**f**) *SRSF2*, (**g**) *TET2*, (**h**) *SETBP1*, (**i**) *NRAS*, (**j**) *JAK2* and (**k**) *EZH2*. The number of evaluable cases for each gene and *P*-value from log-rank test is shown.

The genetic landscape and its prognostic relevance have been explored in CMML.^25, 26, 27, 28^ It is recognized that nonsense and frame-shift mutations of *ASXL1* are adversely prognostic, and the presence of these mutations has now been incorporated in two distinct CMML prognostic models.^14, 15^ As such, we wished to explore the prognostic significance of *ASXL1* and other recurrent genetic mutations in our data set. Because sequence practice patterns were different among contributing institutions, we next confirmed whether our combined data reflected that of published cohorts in the literature. To address this, we identified two cohorts of patients across several institutions that were profiled for more than four clinically significant genes as shown in Figure 5. Encouragingly as expected, mutational frequencies and mutual exclusivities in signaling mutations in these representative subgroups were similar to those reported from other published cohorts.^7, 12, 28^ After confirming this, we explored the prognostic relevance of *ASXL1* (*n*=561), *TET2* (*n*=369), *SRSF2* (*n*=487), *RUNX1* (*n*=377), *EZH2* (*n*=323), *NRAS* (*n*=367), *CBL* (*n*=374) and *JAK2* (*n*=789) in all evaluable cases comprising the most frequently mutated genes in CMML. In the context of 23 clinical variables, we were able to confirm the known prognosis significance of *ASXL1* (*P*<0.0001) and additionally demonstrated that *CBL* (*P*=0.0001) and *RUNX1* (*P*=0.0001) had similar prognostic significance in our data set. After correction for hemoglobin, circulating blasts, platelets and karyotype, we identified *ASXL1* (*P*=0.0114) and *CBL* (*P*=0.003) mutations as independently prognostic (Supplementary Table S3).

We also explored the relative prognostic power of existing CMML clinical models compared with those with *ASXL1* mutation using the previously used ROC and C-index approach. We identified 298 cases for which data on all prognostic models and *ASXL1* mutation were available. These cases were similar in WHO and French–American–British subtype to the larger CMML cohort (Supplementary Table S4). However, no statistical difference in those models containing *ASXL1* mutation was identified compared with models containing clinical variables alone (Supplementary Figure S3).

## Discussion

CMML is a rare hematologic neoplasm that has been confirmed to be distinctly different from MDS. However, much of standardization in CMML clinical practice remains based on the MDS data partially because, unlike MDS, large CMML data sets have not been available to generate evidence-based clinical recommendations. Our data set represents the largest international CMML-specific collection. This provided us sufficient resolution to accurately estimate frequencies of key clinical characteristics and interrogate the utility of existing CMML prognostic models. Of particular interest, our data demonstrated that the majority of CMML cases are CMML-1 (BM blasts <10%) by the WHO classification schema. We were able to demonstrate that a cut-point of 7.5% BM blasts may provide improved prognostication, as cases with 5–9% blasts had a similar OS compared with those with >10% blasts. The adverse prognosis associated with CMML cases with 5–9% BM blasts has been substantiated by a recent publication from the Dusseldorf registry, which was not part of this data set.^29^ A new BM blast cut-point should therefore be validated under central pathology review and subsequently be considered as a revision to the current CMML classification schema.

Our data set also allowed us to validate seven distinct prognostic models used in daily CMML clinical practice. Although all models were valid, it is notable that the prognostic significance of the IPSS and R-IPSS were valid in our entire data set because proliferative CMML cases were excluded in the development of both the IPSS and R-IPSS. Further, even when only proliferative CMML cases (MPN-CMML) were considered, the R-IPSS and IPSS remained valid, albeit with decreased prognostic power as measured by AUC and C-index (Figure 2).

We performed a detailed statistical analysis to compare the relative prognostic power of existing CMML clinical models using the ROC and C-index. We also devised a ‘vulnerability score' to determine the stability of low-risk CMML cases for each model. Although we hypothesized that these analyses would yield a statistically superior model that could be used as a consensus model for future CMML prognostication, we found that all models performed modestly but are insufficiently powerful because all low-risk groups were vulnerable to ‘upstaging.'

Therefore, we attempted to create a novel model using the random survival forest approach. We reasoned that a novel method for variable discovery may uncover uniquely prognostic variables missed by traditional Cox proportional hazard regression. However, our new model generated with this approach had comparable performance when statistically analyzed in the context of existing prognostic tools. Taken together, our data suggest that the prognostic power of clinical variables may have reached an asymptote and that novel prognostication strategies are needed to accurately estimate the OS of patients with CMML. To address this, we explored the prognostic impact of genetic data retrospectively annotated in our database. Although this genetic information was not centrally collected, we were able to demonstrate that frequencies, mutual exclusivities and the expected prognostic relevance of *ASXL1* were maintained, supporting the use of this data set for future study. We were also able to identify the independent prognostic significance of *CBL* mutations in CMML, which had not previously been demonstrated. This is relevant given that our analysis exploring the relative prognostic power of models containing *ASXL1* mutations identified no difference in prognostic power compared with other models, perhaps suggesting that combinations of mutations such as *CBL* and interrogation of RNA expression signatures may yield a more powerful molecular prognostic model. This strategy has been fruitful in other related hematologic malignancies.^30, 31, 32^

It is important to note that this data set was retrospective, included hypomethylating agent-treated cases and did not uniformly capture cases at diagnosis secondary to differing referral patterns. Although the majority of cases had one gene molecularly profiled, 298 annotated cases were used to test the prognostic power of *ASXL1* models. A larger molecularly annotated data set is required to validate our findings. However, the current data set reflects a ‘real-world' collection of CMML cases that could be reliably used to validate future biomarkers. Efforts are now ongoing to further populate this data set with molecular data and operationalize a Web-based portal by which the CMML community can leverage this resource.
