# Supplementary material for: An international data set for CMML validates prognostic scoring systems and demonstrates a need for novel prognostication strategies
Source: Blood Cancer J. 2015 Jul 31;5(7):e333–. doi: 10.1038/bcj.2015.53 (PMC4526779; doi:10.1038/bcj.2015.53)
Supplement: Supplementary Tables 1 and 2 [file bcj201553x1.docx]

**Table 1.** Overall and Center Specific Baseline Characteristics for the International CMML database.

| **Variable (%)** | **Moffitt** | **Mayo** | **MDA** | **GFM** | **CCF** | **Milan** | **RBH** | **MSK** | **Overall** | |
| --- | --- | --- | --- | --- | --- | --- | --- | --- | --- | --- |
| Male | 67 | 76 | 68 | 68 | 66 | 67 | 55.8 | 67 | | **73** |
| Favorable Karyotype IPSS* | 71 | 70 | 63 | 77 | 63 | 73 | 80.5 | 76 | | **72** |
| Favorable Karyotype CPSS * | 68.3 | 68.5 | 61 | 74 | 61 | 69.3 | 80.5 | 76 | | **71** |
| Favorable Karyotype Mayo * | 68.6 | 68.5 | 61.1 | 73.9 | 61 | 68.5 | 80.5 | 76.4 | | **71** |
| WHO CMML-1 | 83 | 84 | 70 | 83 | 83 | 88 | 87 | 83 | | **83** |
| FAB MPN-CMML | 59.6 | 50.4 | 51 | 36.5 | 42 | 44 | 54 | 72.7 | | **51** |
| Spleen^ | 34 | 27 | 22.2 | 11.6 | 24 | 33 | 17 | 51 | | **27** |
| Plt Transfusion** | 35 | 7 | 9 | 9 | 11 | 9 | 6.4 | 61 | | **18** |
| RBC Transfusion*** | 49 | 23 | 27 | 16 | 33 | 24 | 45 | 76 | | **37** |
| ECOG 0 | 14 | 46 | 19 | - | 23 | 33 | 35 | 14.5 | | **26** |
| Overall Survival (mo) | 31 | 22.7 | 24 | 64 | 30.2 | 42 | 22 | 34 | | **30** |

Moffitt=Moffitt Cancer Center, Mayo=Mayo Clinic, MDA= MD Anderson, GFM= Groupe Francophone Des Myelodysplasies CCF=Cleveland Clinic, Milan=University of Milan, RBH=Royal Bournmouth Hospital, Memorial Sloan Kettering Cancer Center. *low risk cytogenetics. ^enlarged spleen, **history of platelet transfusion, ***history of red blood cell transfusion.

**Table 2:** Low-risk vulnerability scores for each CMML prognostic model.


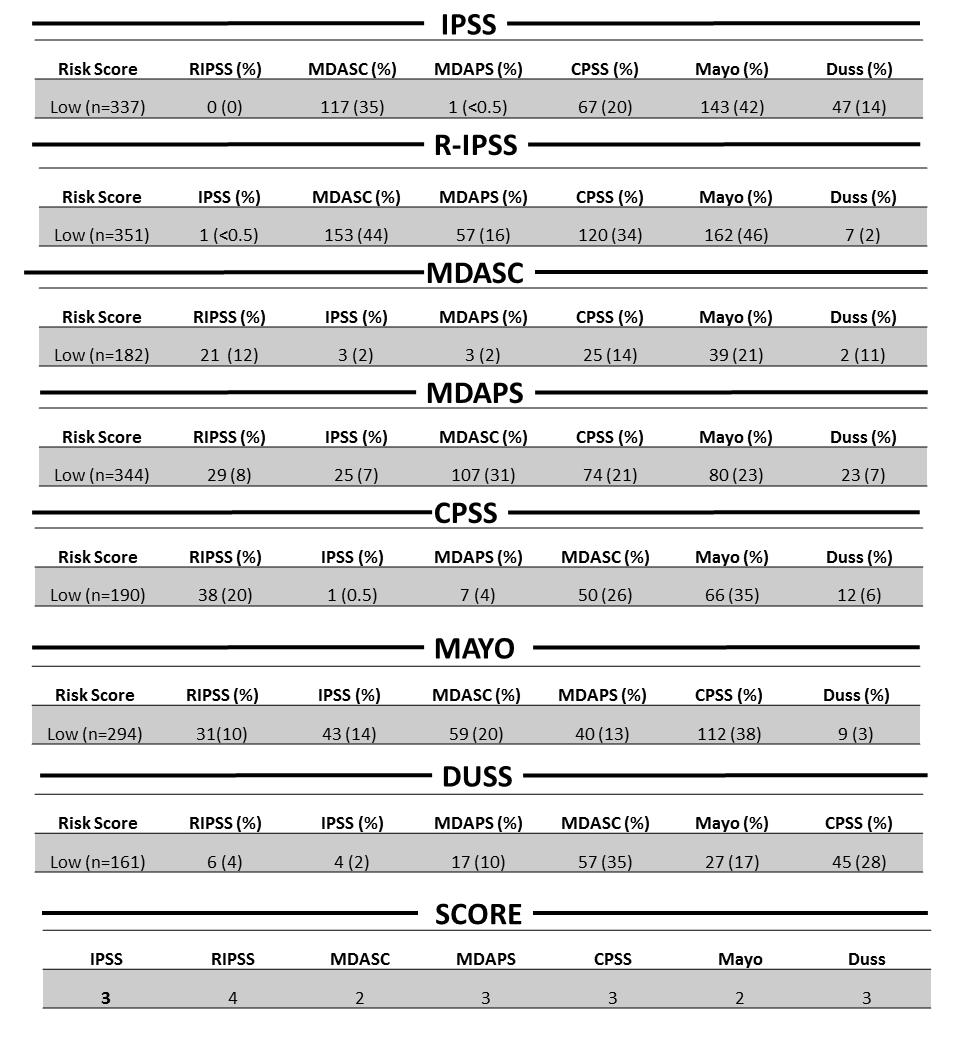


Each low-risk model is represented in the far right. The number and percentage of cases upstaged to higher risk for each competing model is shown. The total vulnerability score is denoted in the last row labeled SCORE.
