# Supplementary material for: An international data set for CMML validates prognostic scoring systems and demonstrates a need for novel prognostication strategies
Source: Blood Cancer J. 2015 Jul 31;5(7):e333–. doi: 10.1038/bcj.2015.53 (PMC4526779; doi:10.1038/bcj.2015.53)
Supplement: Supplementary Table Legends [file bcj201553x4.docx]

Table Legend for Supplementary Table 3:

**Title**: Multivariate analysis for gene mutations that reached univariate significance.

**Legend**: Data demonstrates Uni- and Multi-variate hazard ratios (HR) and p-values for ASXL1 (upper panel), CBL (middle panel), and RUNX1 (bottom panel).

Table Legend for Supplementary Table 4:

**Title**: Base line characteristics comparison between total cohort and genetically annotated cohort.
**Legend**: Data demonstrates WHO categories (CMML-1=1, CMML-2=2), FAB categories (MDS-CMML=1, MPN-CMML=2), and Karyotype by the CPSS model (low risk=1, intermediate risk=2, and high risk=3).
