# Supplementary material for: An international data set for CMML validates prognostic scoring systems and demonstrates a need for novel prognostication strategies
Source: Blood Cancer J. 2015 Jul 31;5(7):e333–. doi: 10.1038/bcj.2015.53 (PMC4526779; doi:10.1038/bcj.2015.53)
Supplement: Supplementary Information [file bcj201553x5.docx]

**Supplementary Figure Legend:**

Figure 1: Kaplan Meier Survival curve of BM blast %. Data is grouped by cases with <5%, 5-9%, or >10% BM blasts.

Figure 2: Kaplan Meier Survival curve of new model derived from variables discovered with the Random Forest Survival Analysis. 1011 evaluable cases were used and long rank test identified a p value <0.0001.

Figure 3: ROC Curves of all existing clinical and genetic models including the GFM^14^ and MMM^15^ models for OS at 36 months. No statistically significant differences were identified in 298 cases in AUC.

**Supplementary Figures:**

**Figure 1:**

**
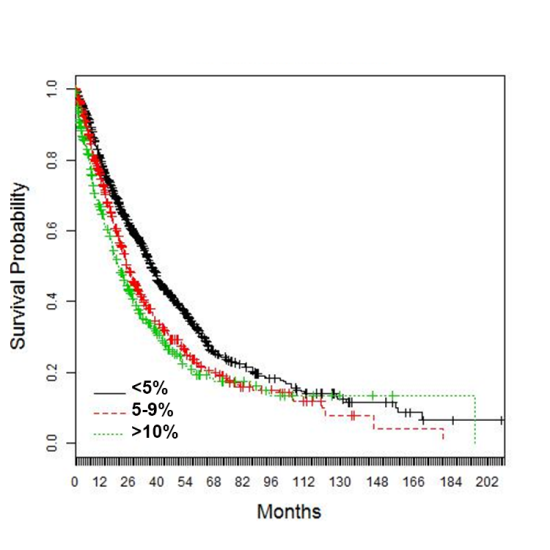
**

P<0.0001

P<0.0001

**Survival Probability**

**Figure 2:**

P<0.0001

P<0.0001

**Figure 3:**
